# Acute Generalized Exanthematous Pustulosis: A Painful and Pruritic Presentation of Pustulosis Following Pharmacotherapy for Pharyngeal Phlegmon

**DOI:** 10.7759/cureus.24072

**Published:** 2022-04-12

**Authors:** Chase Silvers, John Casey

**Affiliations:** 1 Department of Emergency Medicine, OhioHealth Doctors Hospital, Columbus, USA

**Keywords:** acute medical conditions, acute illness, fever with rash, skin eruption, palm and sole rash, diffuse rash, uncommon rash, atypical rash, drug rash, cutaneous drug eruption

## Abstract

Acute generalized exanthematous pustulosis (AGEP) is primarily a drug-induced skin eruption, which typically presents within two days of starting an offending medication; it is often self-limiting with spontaneous resolution within two weeks upon medication cessation. We report the case of a patient who presented with generalized desquamation, characteristic pustules, and several morbilliform rashes on the body surface in association with recent amoxicillin-clavulanic acid exposure. This was associated with significant pruritus, which was the associated chief complaint. A multimodal approach to symptomatic management with topical corticosteroids, barrier ointments, oral antihistamines, and analgesics was required, in addition to the cessation of the offending medication.

## Introduction

Acute generalized exanthematous pustulosis (AGEP) is a painful and pruritic drug-induced skin eruption presenting with sterile pustules on an erythematous base with subsequent desquamation. This uncommon dermatological disease is estimated to affect one in every three to five million patients but is thought to be underreported [1]. Of note, 90% of cases are associated with starting an offending medication, and the onset is typically within two days; it usually resolves within two weeks upon the discontinuation of the drug [1]. Common offending agents include but are not limited to penicillins, hydroxychloroquine, calcium channel blockers, carbamazepine, sulfonamides, and tetracyclines [1-2]. Additionally, several viral infections (Epstein-Barr, Cytomegalovirus, hepatitis B, enteroviruses, etc.) and even spider bites have been implicated as etiologies [1-2]. The initial differential diagnosis for the emergency clinician in the absence of a clear presentation or clear association with a common medication would include more severe illnesses including Stevens-Johnson syndrome, toxic epidermal necrolysis, erythema multiforme, and a broad range of infectious etiologies as the condition can present as erythematous lesions with systemic symptoms initially [2-3]. Drug rash with eosinophilia and systemic symptoms (DRESS) can present similarly but is usually associated with more prolonged medication exposure over weeks [1]. We present a case of AGEP associated with significant pruritus as the presenting chief complaint in the setting of generalized desquamation following the acute phase of amoxicillin-clavulanic acid-induced skin eruption, as well as its associated management.

## Case presentation

A 63-year-old male with a history of emphysema, eczema, gout, diabetes mellitus, obstructive sleep apnea, coronary artery disease, and hyperlipidemia presented to the emergency department with the chief complaint of a painful and pruritic rash. The symptom onset had been seven days prior to the presentation. The rash had erupted on the arm and inguinal folds and progressed to the trunk, extremities, and scalp. On arrival, his main complaint was intense pruritus and skin dryness. Two weeks prior, the patient had been admitted to the hospital for a deep-space soft tissue infection of the neck and had been treated nonoperatively with oral antibiotics (amoxicillin-clavulanic acid) for 14 days. He endorsed a near-resolution of the neck swelling and discomfort associated with the soft tissue infection. When presenting to the emergency department, he was on his last day of the prescribed antibiotic course. The symptom onset had been approximately one week after the initiation of the antibiotic with the development of a rash. Subsequent dryness and flakiness of the skin had developed three days prior to the arrival. The patient reported compliance with home medications and denied any other new treatments, environmental exposures, or insect bites. He affirmed no drug allergies but admitted to seasonal allergies and a history of eczema without any history of psoriasis. The patient endorsed and affirmed no concerns about sexually transmitted infections. He denied alcohol, recreational drug, or tobacco use.

He had no complaint of eye pain or redness, tongue swelling, fevers, or sores of the mouth, anus, or genitalia. The remainder of the review of the systems was otherwise negative. His chronic home medications included allopurinol, albuterol inhaler, aspirin, atorvastatin, cetirizine, clopidogrel, furosemide, lisinopril, metoprolol succinate, montelukast, and nitroglycerin.

**Figure 1:**
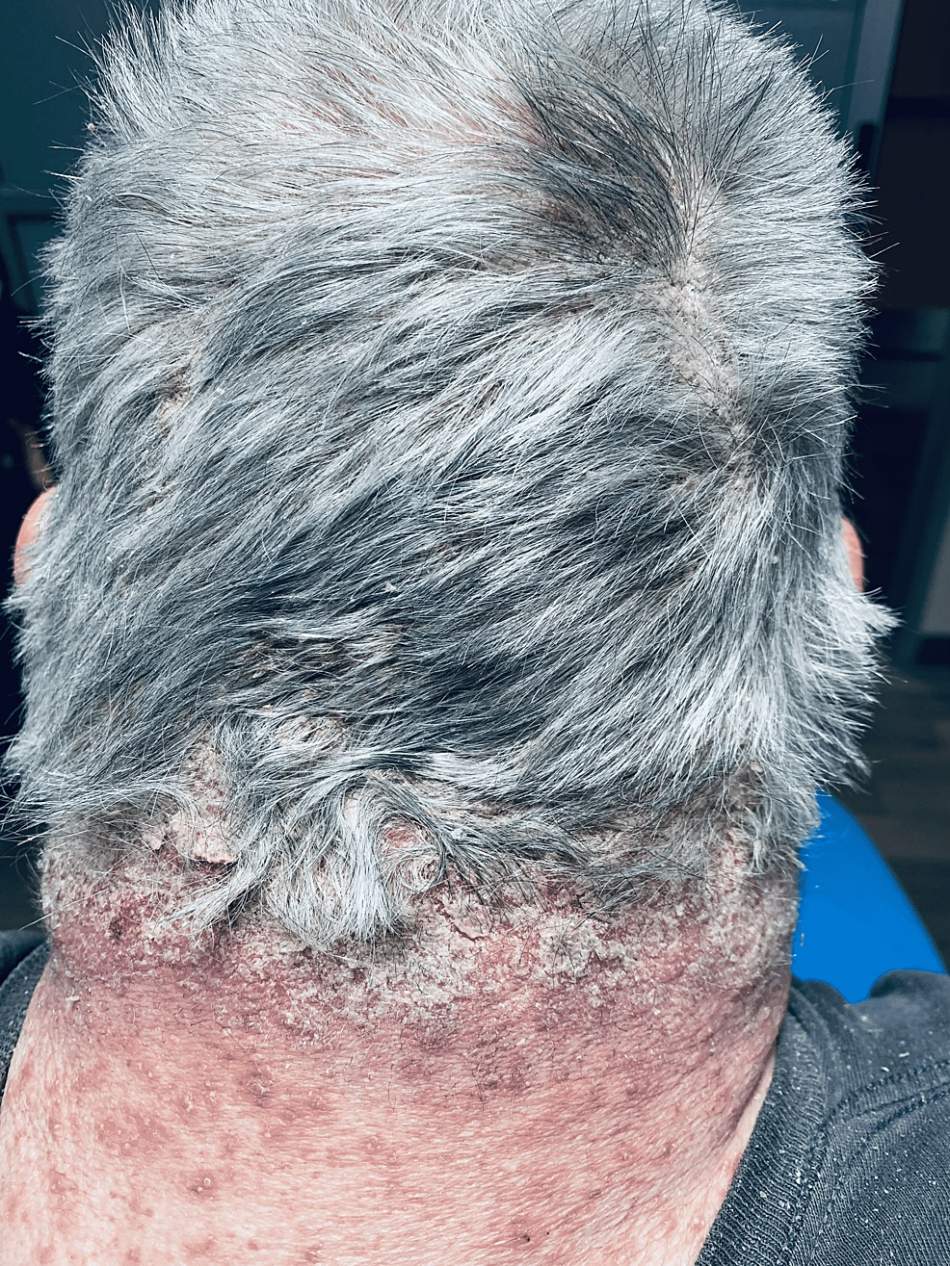
Erythematous and morbilliform rash with deroofed pustules of the neck and extensive desquamation of the scalp

**Figure 2:**
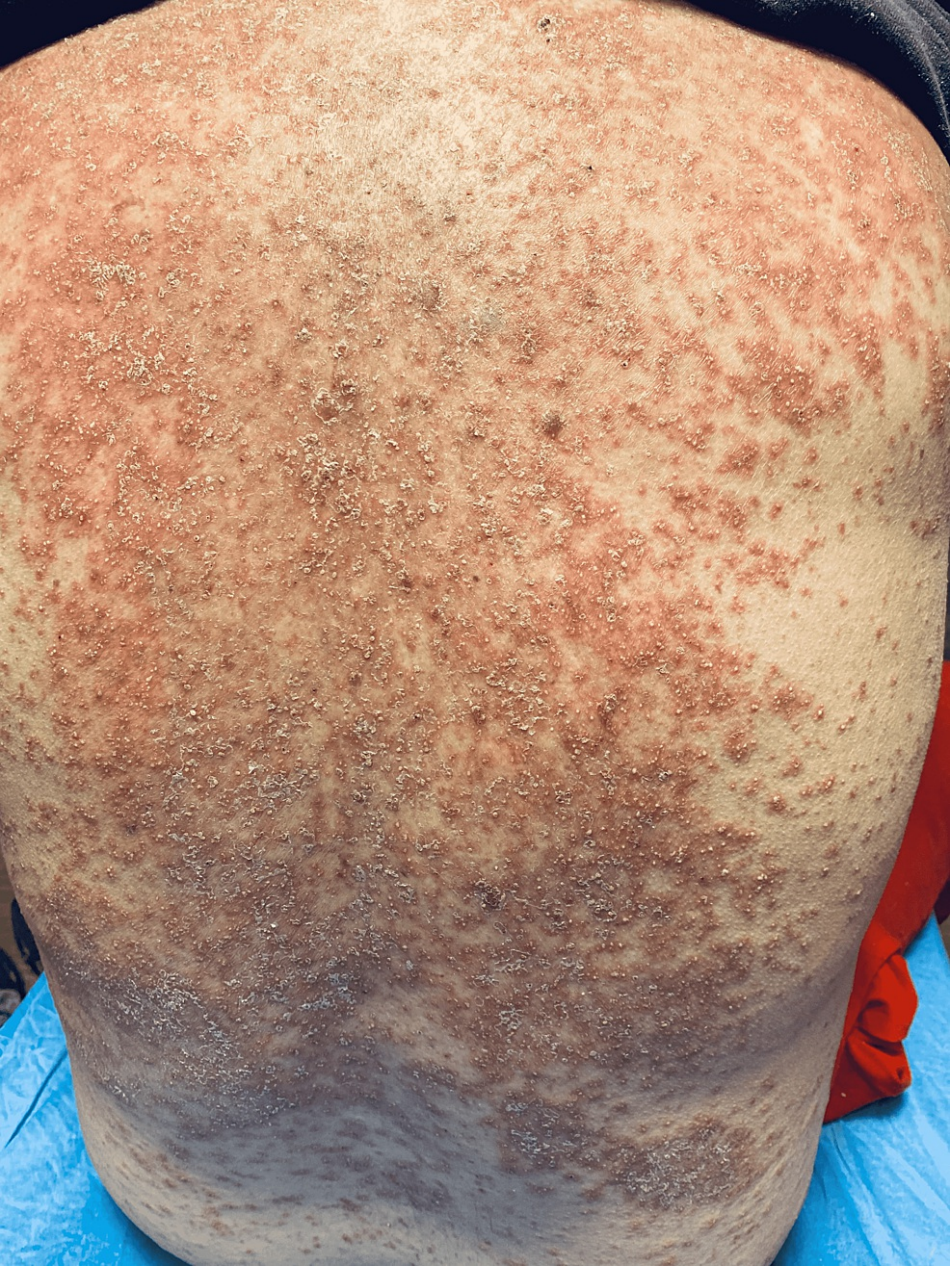
Erythematous and morbilliform rash with pustules and desquamation on the back

**Figure 3:**
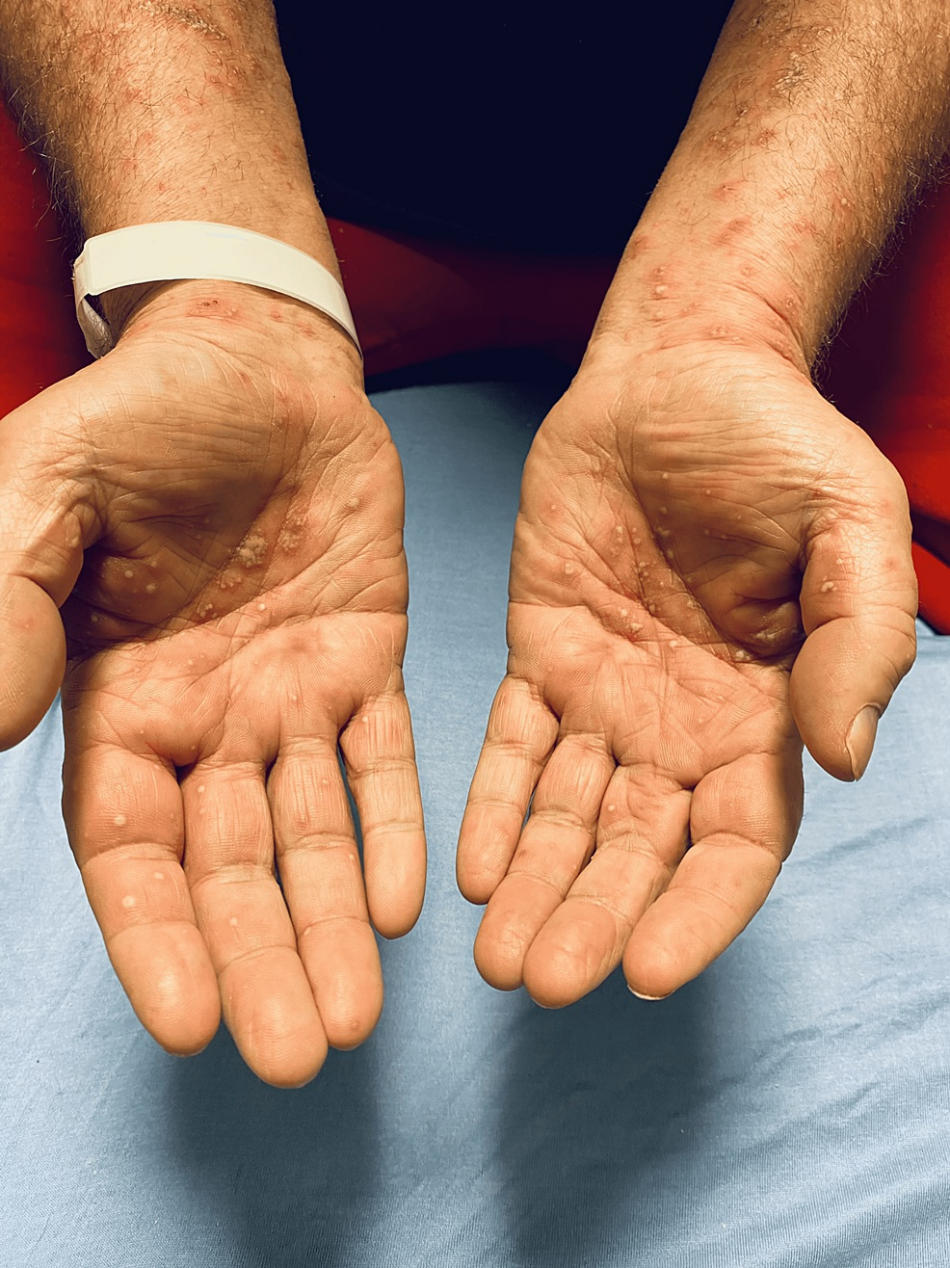
Erythematous and morbilliform rash with pustules affecting upper extremities including palms

The patient's vital signs were as follows: blood pressure of 155/68 mmHg, heart rate of 108 beats per minute, respirations of 16 breaths per minute, oral temperature of 97.7 °F, and 97% pulse oximetry on room air. The physical exam was notable for a diffuse, generalized, erythematous, and desquamating rash involving multiple body surfaces as shown in Figures 1-3. These were associated with pin-point pustules of the trunk, extremities, scalp, and palms, but sparing the soles of feet, as depicted in Figures 2, 3. Nikolsky's sign was negative. The affected skin was warm and dry without palpable induration. There was no evidence of mucosal involvement in the oral, ocular, or genitorectal body regions. The rest of the physical exam was otherwise non-contributory.

Previous laboratory investigations from two weeks prior were pertinent for a leukocytosis of 13,000 K/mcL without differential, normal creatinine of 0.95 mg/dL, within normal limit electrolytes, and stable baseline normocytic anemia.

Given the recent antibiotic exposure, characteristic skin findings, and the absence of systemic symptoms and mucosal involvement, along with the suspicion of a drug eruption, AGEP was primary on the differential. Dermatology was contacted and they recommended symptomatic treatment with topical steroids of moderate potency, barrier ointments, and antihistamines for pruritus until follow-up in their clinic within the week. The patient’s symptoms were controlled and he was provided with strict return precautions. The patient was ultimately lost to follow-up and was found to have had no return visits upon review of the electronic medical records at one and three months following the initial encounter.

## Discussion

AGEP is an uncommon skin eruption with multiple etiologies and diagnoses to consider. It is occasionally associated with systemic symptoms and more severe illnesses [1]. Skin pustules may not be observable in the first days of illness and may present as erythematous or morbilliform in appearance [2]. Mortality associated with AGEP is less than 5% and is generally linked to underlying comorbidities [1]. Some patients may present with fever and generally feel unwell, but many others may be asymptomatic except for the presence of rashes [2]. Common laboratory abnormalities include neutrophilia, eosinophilia, and elevated inflammatory markers (elevated sedimentation rate and C-reactive protein) [2]. During the acute presentation of illness, some patients may be admitted for observation or may have a close outpatient follow-up if symptoms are minimal and controlled.

We discussed a case of AGEP following the initial acute phase with a primary complaint of significant pruritus and flakiness of skin without systemic symptoms while taking amoxicillin-clavulanic acid for an improving pharyngeal soft tissue infection. Initial management of the condition typically consists of discontinuing the suspected medication [1-2,4]. Subsequent therapies entail symptomatic control, including topical corticosteroids, oral analgesics, and antihistamines [1-2,4]. Systemic corticosteroids are rarely necessary, given the self-limiting nature of the illness. However, one retrospective study reported a decreased length of stay among hospitalized patients without adverse events and no difference in secondary outcomes compared to those treated topically [4]. One study has identified systemic corticosteroids as a cause of AGEP [5]. There have been reports of expedited resolution with immunomodulators such as cyclosporine [6]. Patients should be urgently referred to a dermatologist for skin biopsy and further monitoring to evaluate for alternative pustular eruptions of significance [3]. Patch testing within six months of the event can be performed in order to identify the causative agent so that recurrence can be prevented [7].

## Conclusions

AGEP is primarily a drug-induced skin eruption that typically develops within two days of starting an offending medication. It is usually self-limiting with spontaneous resolution within two weeks upon medication withdrawal. Subsequent management aims to deal with more severe illnesses if the initial diagnosis is unclear or symptomatic control is unsuccessful. Patients may experience significant pruritus and skin irritation after the acute phase when desquamation occurs. Topical corticosteroids, barrier ointments, oral analgesics, and antihistamines are the mainstays of therapy.
